# Testing the feasibility of augmented digital skin imaging to objectively compare the efficacy of topical treatments for radiodermatitis

**DOI:** 10.1371/journal.pone.0218018

**Published:** 2019-06-10

**Authors:** Richard Partl, Jörg Lehner, Peter Winkler, Karin Sigrid Kapp

**Affiliations:** Department of Therapeutic Radiology and Oncology, Medical University Graz, Comprehensive Cancer Center Graz, Graz, Austria; University of Arkansas for Medical Sciences College of Pharmacy, UNITED STATES

## Abstract

**Introduction:**

Radiation-induced dermatitis (RID) is routinely graded by visual inspection. Inter-observer variability makes this approach inadequate for an objective assessment of the efficacy of different topical treatments. In this study we report on the first clinical application of a new image-analysis tool developed to measure the relevant effects quantitatively and to compare the effects of two different topical preparations used to treat RID.

**Materials and methods:**

After completion of radiotherapy, RID was retrospectively assessed in 100 white female breast cancer patients who had received adjuvant breast irradiation. Of these patients, 34 were treated with R1&R2, a Lactokine-fluid derived from milk proteins, and 66 were treated with Bepanthen. In addition RID was graded independently by two experienced radiation oncologists in accordance with the Common Terminology Criteria for Adverse Events (CTCAE). For quantitative evaluation, the irradiated breast and the non-irradiated contralateral breast were photographed in a standardized manner including a color reference card. For analysis, all images were converted into the color space L*a*b* and mean values were calculated for each of the color parameters.

**Results:**

The CTCAE-based grading revealed statistically significant inter-observer variability in the scoring of RID Grades 1, 2 and 3 (p<0.001). A difference between the two topical products could not be observed with visual inspection. By using augmented image analysis methods a statistically significant increase in a*-values (mean 4.15; 95%CI: 5.97–2.33, p<0.001) in patients treated with R1&R2 indicated more intense reddening. Digital subtraction was used to eliminate differences in individual baseline skin tone to generate a new, low-scatter parameter (ΔSEV).

**Conclusions:**

Visual CTCAE-based evaluation of RID was not suitable for assessing the efficacy of the skin treatment products. In contrast, the novel image analysis enabled a quantitative evaluation independent of skin type and baseline skin tone in our cohort suggesting that augmented image analysis may be a suitable tool for this type of investigation. Prospective studies are needed to validate our findings.

## Introduction

Acute radiation-induced dermatitis (RID) occurs in 95% of patients in whom the skin is part of the radiation target [1]. Most affected are patients with breast cancer, head and neck tumors and patients who need high dosage to inguinal nodes, genitals or perineum. The severity of the RID varies depending on dose per fraction, total dose, beam quality, radiation technique, prior chemotherapy, and skin type.

Although there is a widely acknowledged need for objective and automated measurement and documentation of therapy-related RID, no practicable tools have been available for routine clinical use until now. Currently, RID is still evaluated using subjective visual classifications such as those provided by the Common Terminology Criteria for Adverse Events (CTCAE) and the Radiation Therapy Oncology Group/European Organisation for Research and Treatment of Cancer (RTOG/EORTC) [2,3]. Even the definition of CTCAE Grade 1, ‘faint erythema’ already gives the observer considerable room for interpretation; and where is the transition to ‘moderate erythema’ (Grade 2)? This inevitably leads to a non-negligible inter-observer variability. Another problem is the variety of qualitatively different skin changes which can be summarized under the same toxicity grade.

To avoid this bias, there is a need for new sensitive, objective and economical tools that enable measurement and documentation of skin changes and which are convenient enough to be used in routine clinical practice. Such tools would also expedite the search for more effective treatments of RID.

In several publications, a subjective assessment of RID was complemented by using instruments to measure skin color, skin hydration or dermal oxygenation of hemoglobin [4–7] with methods that measure patches of skin only a few millimeters across, which is problematic because irradiated skin reacts inhomogenously on this scale, depending for example on skin thickness, skin folds, mechanical stress; to estimate RID accurately, an evaluation of the whole irradiated area is needed. Chin et al. [8] used a spectrophotometric device for imaging defined areas within the irradiated breast controlling for the non-irradiated breast before and after each treatment fraction and observed a strong correlation between changes in oxygenated hemoglobin and skin reaction as well as between radiation exposure and changes in skin reaction.

In our study we used a commercially available digital single-lens reflex camera to perform augmented digital imaging of the entire region of the irradiated and non-irradiated breast. Spectrophotometric color measurements of L* (lightness value) and a* (red-green component) in the CIELAB color space [9] have already been recommended for evaluation of RID by other authors [5,10,11]. A recent study showed by means of in-silico analysis that the a* value alone is not a reliable parameter. This means that lighter and darker red skin locations can return the same a* values–and this is a realistic scenario in RID. Applying the algorithm (L*max–L*) x a* a standard erythema value (SEV) can be calculated to overcome this weakness. By analyzing high-resolution image data on the scale of several megapixels the quality of erythema assessment could be increased in such a magnitude that for the first time cycling erythema waves became visible on a single patient level. The method has been validated with spectrophotometric single point measurements and revealed superiority to all previous approaches. Especially in cases when skin reactions in representative areas appeared inhomogeneous or visual differences in the erythema were low [12].

In this proof of concept study, the augmented digital skin imaging method is clinically tested for the first time to evaluate the feasibility to objectively compare two different topical products which are used to treat RID at our center.

## Materials and methods

### Study design

100 consecutive white female patients referred for adjuvant radiotherapy following breast conserving surgery for early unilateral breast cancer without known allergy to the components of the two skin treatments employed were retrospectively analysed. The products tested were: R1&R2 (Water-Jel Technologies), which contains Lactokine-fluid, derived from milk proteins [13] and Bepanthen (Bayer), which contains the active ingredient pro-vitamin Dexapanthenol [14], that is widely used in dermatologic therapy.

The irradiation of the affected breast was carried out using a 3D conformal target-volume technique with 6 MV photons. Depending on the breast volume, the total dose was 50.0–50.4 Gy and was applied in 25 or 28 fractions 5 days per week. The irradiated area was treated topically, from the first day of irradiation onwards, with R1&R2 in 34 patients, and with Bepanthen in 66, respectively.

This study was approved by the ethics committee of our Medical University (Protocol Number: 31–050 ex 18/19) and was conducted according to Good Clinical Practice guidelines and the Declaration of Helsinki.

### Image acquisition and data processing

The irradiated area and the non-irradiated contralateral breast were both photographed immediately after the completion of radiotherapy using a digital single-lens reflex camera (Canon EOS 500D). The photographs were taken in uniform lighting with standardized camera settings and saved in RAW format.

**Fig 1:**
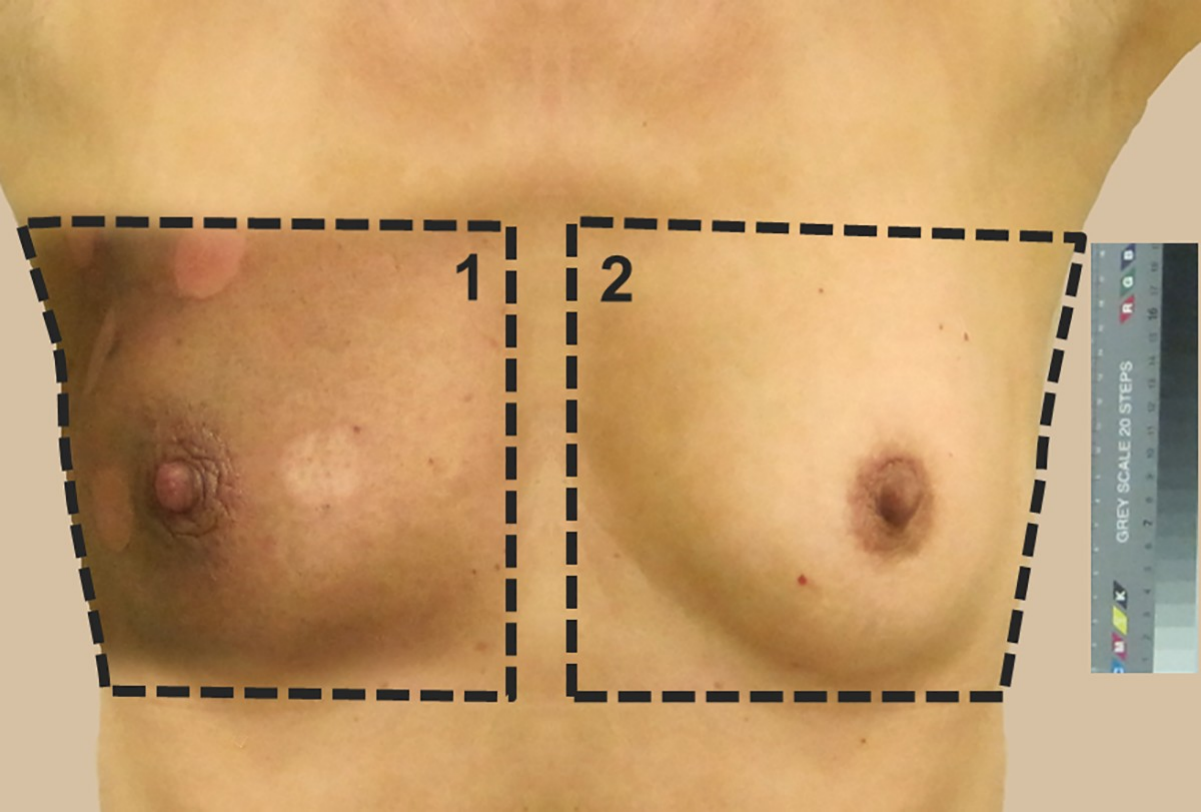
Digital photographs of the measurement areas 1 (irradiated skin) and 2 (reference area on contralateral breast) under standardized lighting and with calibration of the images using a color reference card.

The images were color calibrated using a color reference card and the areas to be measured were defined. Measurement area 1 comprised the whole irradiated area; measurement area 2 was the non-irradiated contralateral breast as a control (Fig 1). Each of these measurement areas had a minimum data volume of 10^6^ pixels. For analysis, the pixels were converted from the RGB color space to the L*a*b* (CIELAB) color space using the software Matlab (Release 8.1, 2013, MathWorks, Inc.). In the last calculation step, mean L*a*b* values were calculated for the whole measurement areas of the experimental and control images (S1 Attachment). The L*a*b* color space is approved by the French Commission Internationale de l´Éclairage (CIE) [9] and is an industry standard [15] that describes all perceptible colors in a device-independent manner and which models human color perception. Each color in the color space is represented by a color position with the Cartesian coordinates {L*, a*, b*}. The a* parameter describes a red-green axis from −170 (green) to +100 (red). The b* parameter describes a blue-yellow axis from −100 (blue) to +150 (yellow). L* is a lightness scale, from the darkest black at L* = 0 to white at L* = 100, containing all gray tones.

### Visual assessment

The visual evaluation of skin type was done routinely before the beginning of radiotherapy using the 6-point Fitzpatrick Scale [16]. The RID was graded by two experienced radiation oncologists, independently of one another, at the end of radiotherapy and was classified according to CTCAE v4.03. All analyses in this study refer to the toxicity grade after the completion of radiotherapy.

### Statistical data analysis

For continuous data, mean and standard deviation or mean and range were calculated, and results were compared using t-tests, for multiple comparisons analyses of variance and post-hoc tests were used. For categorical data, absolute and relative frequencies were calculated and results were compared using the chi-square test. Statistical analysis was done using IBM SPSS Statistics (Release 23.0.0 2015, Chicago, IL). Statistical significance was defined as a value of p<0.05.

## Results

The median age of the 100 patients at the beginning of radiotherapy was 62.1 years (range: 36.5–80.8). The most frequently observed skin types were Type 3 (60%), Type 2 (32%) and Type 4 (8%) on the Fitzpatrick scale. The mean CIELAB color parameters calculated from the control images were L* = 66.66 (SD: ±6.99), a* = 6.91 (SD: ±3.16) and b* = 16.53 (SD: ±4.75). Patient characteristics are given in Table 1. Both the R1&R2 and Bepanthen treatment groups did not display any significant differences in terms of age, skin type, CIELAB color space of the untreated skin, tumor stage, prior and concomitant systemic treatments.

**Table 1:** Patient characteristics and univariate comparison between treatment groups.

Parameter	Overall value (n = 100)	Bepanthen (n = 66)	R1&R2 (n = 34)	p-value*
**Median age, years (range)**	62.1 (36.5–80.8)	62.1 (36.5–80.5)	62.2 (39.8–80.9)	0.51
**Skin Type, Fitzpatrick scale**				0.92
2	32	22 (33.3%)	10 (29.4%)	
3	60	39 (59.1%)	21 (61.8%)	
4	8	5 (7.6%)	3 (8.8%)	
**Mean CIELAB (±SD) of untreated skin**				
L*	66.66 (±6.99)	66.74 (±6.43)	66.5 (±8.05)	0.87
a*	6.91 (±3.16)	6.57 (±3.12)	7.56 (±3.16)	0.13
b*	16.53 (±4.75)	16.22 (±4.62)	17.11 (±5.01)	0.37
**Tumor stage**				0.75
1	68	46 (69.7%)	22 (64.7%)	
2	28	17 (25.8%)	11 (32.4%)	
3	4	3 (4.5%)	1 (2.9%)	
**Prior chemotherapy**				0.69
yes	27	17 (25.8%)	10 (29.4%)	
no	73	49 (74.2%)	24 (70.6%)	
**Endocrine therapy**				0.67
yes	80	52 (78.8%)	28 (82.3%)	
no	20	14(21.2%)	6 (17.7%)	
**Breast cup size**				0.06
A	11	7 (10.6%)	4 (11.8%)	
B	42	34 (51.5%)	8 (23.5%)	
C	30	17 (25.8%)	13 (38.2%)	
D	13	7 (10.6%)	6 (17.7%)	
E	4	1 (1.5%)	3 (8.8%)	

CIELAB, color space by the French Commission Internationale de l'Éclairage; L*, represents the lightness; a*, the green–red color components; b*, the blue–yellow components; SD, standard deviation.

* p-values were calculated using t-test or chi-square test.

### Inter-observer variability

**Fig 2:**
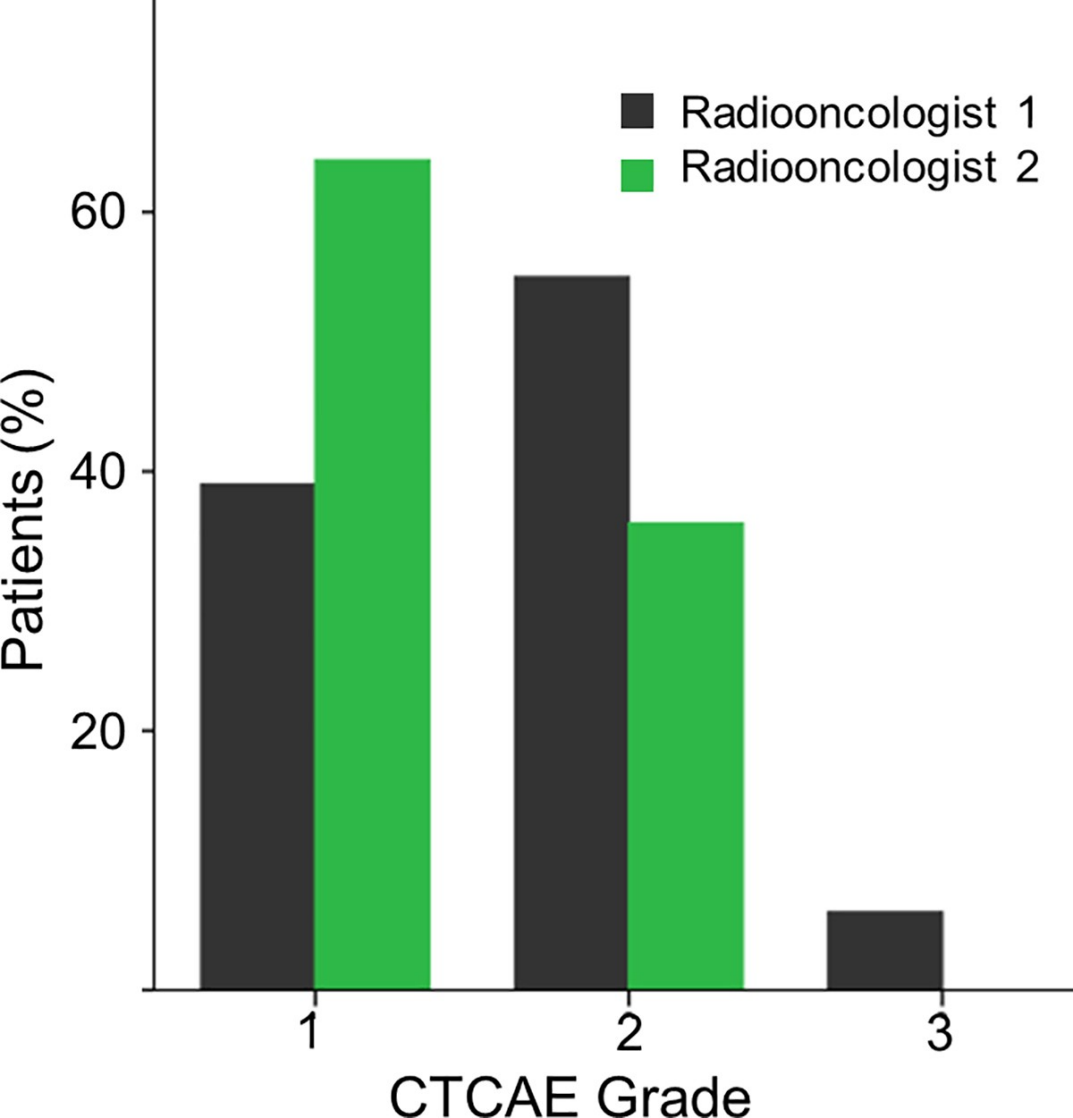
Grading of acute skin toxicity according to CTCAE 4.03 by two radiation oncologists independently of each other exhibited significant inter-observer variability (p<0.001).

The assessment of RID based on CTCAE by two board-certified radiation oncolologists revealed statistically significant inter-observer variability (p<0.001), (Fig 2). At the end of radiotherapy, radiation oncologist 1 and radiation oncologist 2 classified 39% v. 64% of patients as Grade 1, 55% v. 36% as Grade 2 and 6% v. 0% as Grade 3. The evaluation of efficacy of the two treatments by this method was inconclusive: Radiation oncologist 1 found no significant difference between the treatments in terms of CTCAE Grades (p<0.053), but radiation oncologist 2 found a reduction of Grade 2 toxicity with R1&R2 (p<0.021).

### Assessment of radiation-induced skin changes

**Fig 3:**
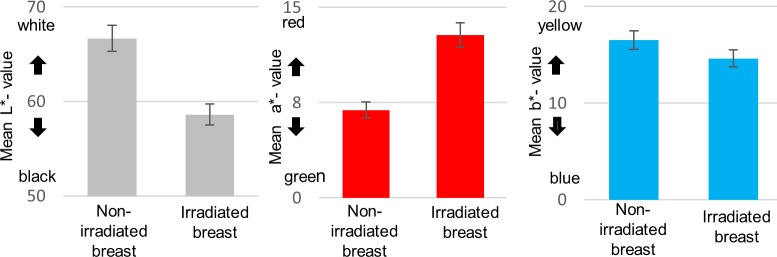
Color measurements showed significant differences of the mean L*a*b* values (p<0.001) between irradiated skin (measurement area 1) and not irradiated control (measurement area 2). Error bars show ± 1 standard deviation (SD).

The color measurements of the skin after radiotherapy (Fig 3) revealed significant changes, relative to the untreated contralateral breast, in the parameters of mean L* (66.66 v. 58.60, p<0.001), mean a* (6.91 v. 12.83, p<0.001), and mean b* (16.53 vs. 14.63, p<0.001). In the clinical context, the reduction of the L* values reflects an increase in pigmentation, the increase in a* values reflects an increase in redness and the reduction in b* values indicate a shift towards the blue end of this scale.

#### Quantitative assessment of the efficacy of R1&R2 compared to bepanthen

**Fig 4:**
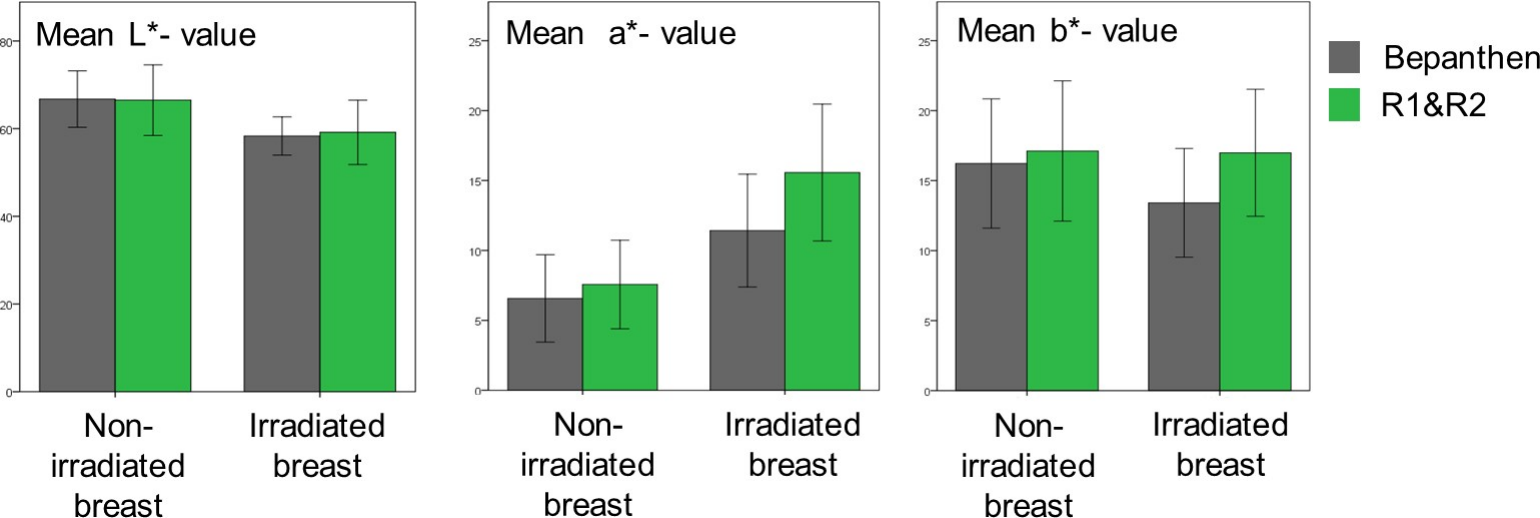
Skin treated with Bepanthen exhibited less redness than the skin treated with R1&R2 (a* = 11.4 ± 4.0 (SD) vs. 15.6 ± 4.9 (SD), p<0.001), but a drop in the yellow component (b* = 13.4 ±3.9 vs. 17.0 ± 4.5, p<0.001). There was no difference in L* values between the treatments. Error bars show ± 1 standard deviation (SD).

After the completion of radiotherapy, the skin changes in patients treated with R1&R2 or Bepanthen were compared quantitatively in the L*a*b* color space (Fig 4). The skin treated with R1&R2 exhibited significantly more redness, by a mean of a* = +4.15 (95%CI: 5.97–2.33, p<0.001). The b* value did not change significantly compared to the untreated breast. Skin treated with Bepanthen showed a drop in the yellow value by a mean of b* = –3.58 (95%CI: 5.23–1.85, p<0.001) and therefore had a lower value than before radiotherapy. There was no significant difference in the L* values between the two groups (95%CI: -3.17–1.48, p = 0.47).

#### Application of the standard erythema value (SEV) and introduction of a baseline skin tone subtraction

By combining the parameters L* und a* in the formula (L*max–L*) x a*, it was possible to generate a new, sensitive parameter (SEV) which registers a higher signal even at low levels of redness.

Applying the SEV formula revealed that the irradiation led to a highly significant increase in the values with a steeper curve than the individual L*a*b* parameters.

**Fig 5:**
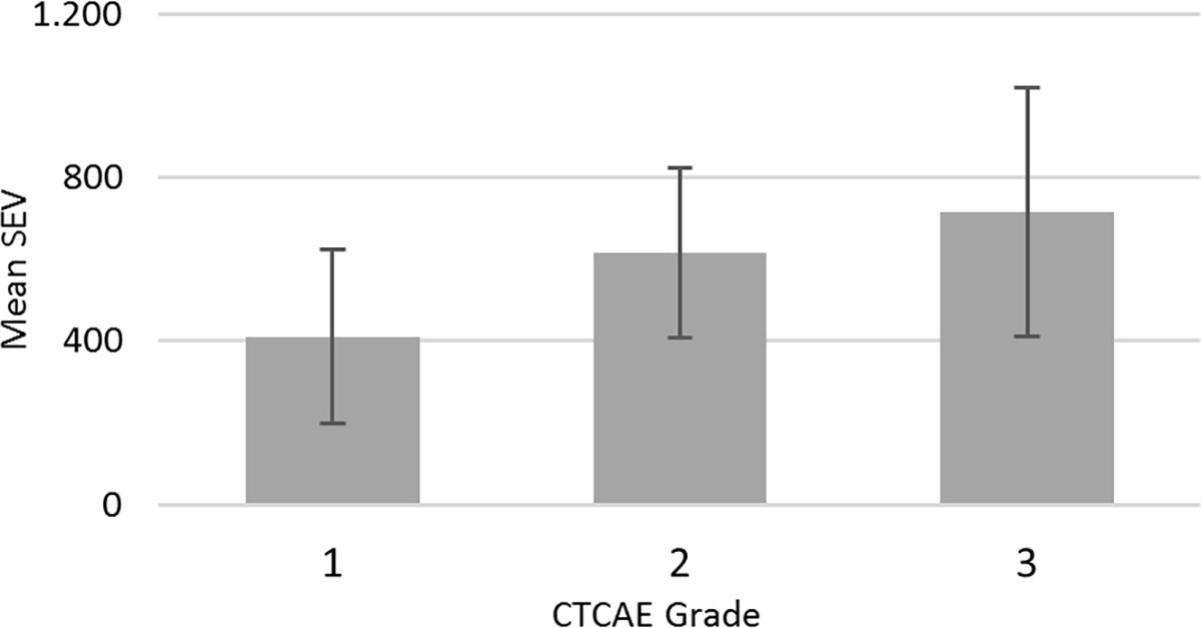
Comparison of CTCAE grade (according to radiation oncologist 1) and the mean SEV values. Error bars show ± 1 standard deviation (SD). Mean difference between CTCAE grade 1 vs. 2 is SEV 205.6 (95%CI: 95.8–315.4; p<0.001), 1 vs. 3 is SEV 306.4 (95%CI: 76.4–536.4; p = 0.005) and 2 vs. 3 is 100.8 (95%CI: -124.7–326.3; p = 0.837).

Patients with RID grade 2 or grade 3 (Fig 5) showed a significant higher SEV value when compared to patients with RID grade 1 (p<0.001; p = 0.005). No significant difference was observed between grade 2 and 3 (p = 0.837).

However, a problem still existed in the form of the individual baseline skin tones of the patients, which caused an unavoidable scatter. A digital subtraction of the baseline skin tone allowed this random error to be removed. The resulting delta SEV (ΔSEV) was a parameter for the color change that was independent of the baseline skin ΔSEV = SEV *(measured area)*–SEV *(baseline color)*. The mean ΔSEV caused by the radiotherapy was 646 (SD: ±48.3).

**Fig 6:**
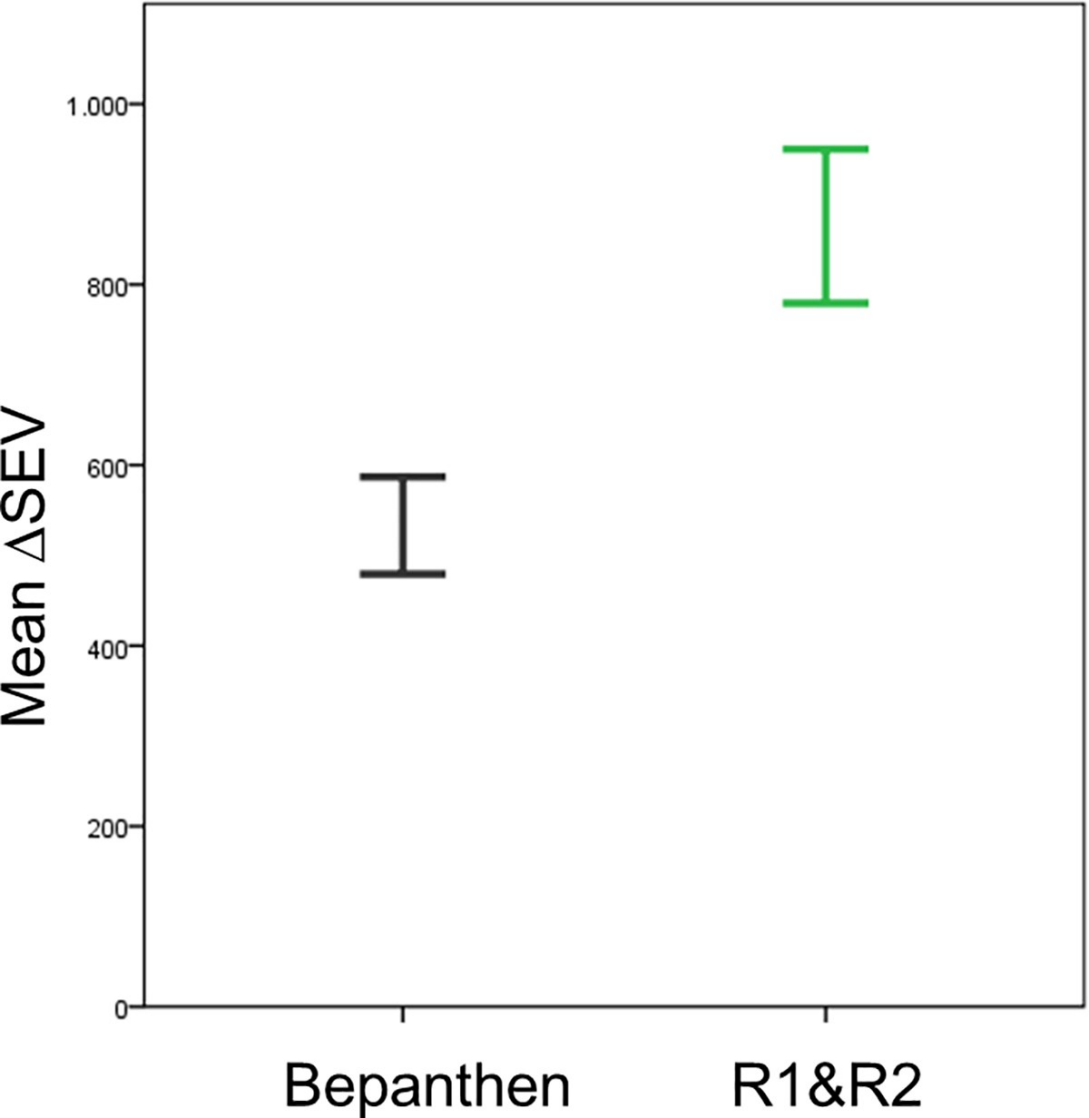
The new parameter ΔSEV showed less change in skin color with Bepanthen than with R1&R2 (p<0.001). Error bars show ± 1 standard deviation (SD).

Also, a significant difference between the two products R1&R2 and Bepanthen was found, with Bepanthen giving better results. The mean color difference between the two products (Fig 6) was 331.5 (p<0.001).

## Discussion

In the early days of radiotherapy RID was actually used as a way to estimate the applied dose, in the form of an ‘erythema dose’ (approx. 5–6 Gy in a single fraction). Although the observed skin erythema did not suffice to define X-ray dosage with satisfactory precision it was the only practicable method of biological measurement in X-ray therapy [17,18]. But of course this has long since been replaced by dosimetry methods since the International X-Ray Unit Committee defined the international unit of radiation, the roentgen, in 1928. In contrast, the grading of RID still relies on purely visual subjective classification systems. These classifications use quite crude scales and carry a considerable risk of inter-observer variability. To estimate the magnitude of this variability, we had the RID grading done according to CTCAE v4.03 by two experienced radiation oncologists working independently of each other. Overall, significant differences were observed (p<0.001). Radiation oncologist 1 identified 52.8% more CTCAE Grade 2 changes than radiation oncologist 2 (n = 55 vs. 36) and even classified 6 patients as Grade 3, but scored 39% fewer patients as Grade 1 (n = 39 vs. 64). The evaluations of radiation oncologist 1 also found no difference between the two treatments used, while radiation oncologist 2 found lower toxicity with R1&R2.

The primary aim of this study was to test our prior validated quantitative method clinically by comparing two different topical treatments in use at our center for RID.

In the in-silico analysis the a* value alone was not a reliable parameter [12]. The reason for this is that a* drops when L* is reduced. In a biological context this means that an increase in erythema leads to an increase in a*; but an increase in pigmentation of the skin leads to a reduction in L* and therefore also to a reduction in a*. This means that lighter and darker red skin locations can return the same a* values–and this is a realistic scenario in RID because increases in pigmentation are observed in response to irradiation in addition to erythema. The SEV overcomes the weakness of single-parameter measurements and is the first objective erythema parameter with a linear response from light to very dark skin (although a limitation of this study is that only white patients were included, because 100% of our patient population is of European descent). By calculating the SEV automatically for every pixel in the digital image, the signal is amplified relative to the spectrophotometric methods by a factor of 10^6^. Using this sensitive method we were recently able, for the first time, to observe erythema waves which correlated to the weekly irradiation cycles. In view of the broad range of possible applications, this technology has already been patented [19].

However, a problem that remained was the variation of the baseline skin tones of the patients, which led to a large degree of scatter of the measured color values. Here we found that digital color subtraction was successful in extracting the ΔSEV as a parameter independent of skin type and skin tone. In order to avoid measurement errors, the measurement areas have to be uniformly illuminated and shiny reflections have to be avoided.

Using this method in our cohort, we were able to show quantitatively that R1&R2 was significantly less effective at reducing RID than Bepanthen. However this is only a proof of concept and the findings should be treated with caution because of a few shortcomings in the study design that have to be taken into account. Having an image acquisition in only one point in time is a limitation. If additional points in time were collected, perhaps early identification of mean L*a*b* values would be clinically more significant. Another shortcoming is the retrospective design that could bear a selection bias. Changes in pigmentation can occur after surgery and chemotherapy that can be an additional confounding factor. If the topical agents were applied in a double-blinded and randomized fashion, the objective method could identify the efficacy of Bepanthen or other topic agents selected for testing more reliable. This study only included white women (Fitzpatrick scale 2–4) so its applicability to populations outside this race are uncertain. Although we could demonstrate with in-silico analyses that the SEV is capable to measure skin reactions ranging from bright to very dark skin tones (Fitzpatrick scale 1 to 6) we lack clinical data for scale 5 (dark brown) and 6 (deeply pigmented dark brown to darkest brown). In order to eliminate these confounders and to draw firm conclusions the feasibility of augmented skin imaging analysis should be validated in further prospective studies.

For quantitative measurement of RID reactions the use of spectrophotometric measurements of the skin color or the oxygenated hemoglobin level have been suggested by previous authors [4–8,10]. The practical advantage of our novel image analysis method over these methods is that no additional hardware equipment is needed. We could demonstrate that the image information of a simple digital camera or a commercial smartphone is suitable to perform objective quantitative measurements. In principle, this clinically convenient and economical method may be a suitable tool for quantitative big data analysis of RID capable to measure both, the efficacy and side effects of investigational topical products and treatment methods. It is assumed being applicable for assessment of time dependent visual changes of skin inflammation or pigmentation irrespective of their etiology and pathogenesis. The necessary steps for the color calibration and the calculations for the Augmented Image Analysis have been incorporated into a mobile app for smartphones. The product is CE certified and is available under the name Scarletred Vision. In addition to RID assessment, Scarletred Vision has recently been used in a broad range of dermatologic applications and skin conditions. For example it has been tested in the assessment of disease activity over time in patients with hidradenitis suppurativa [20], or in the prediction of a treatment response to topical Enstilar foam application in psoriasis [21]. Furthermore it has already been used as a machine learning based system for automated measurement and documentation of wound size, a semi-automatic machine learning psoriasis plaque grading system and an objective method for allergy and substance testing preferentially in the course of pre-clinical and clinical drug development [22].

## Conclusions

The Augmented Digital Skin Imaging Method may present an objective tool for quantitative measurement of skin changes and topical treatment efficacy. It may solve the problem of lacking standardization and objectivity and appears to be a very effective and more sensitive technique than the visual CTCAE grading. We were able to show that a digital color subtraction can be used to generate a parameter for skin changes (ΔSEV) that is independent of the individual baseline skin tone and skin type. This method enables convenient and economical documentation of skin changes irrespective of their etiology and pathogenesis.

## Supporting information

S1 Attachment Data processing for augmented digital image analysis. Image calibration and color space conversion from RGB to CIELAB using Matlab, mean L*a*b* calculation.
(PDF)
