# Supplementary material for: Testing the feasibility of augmented digital skin imaging to objectively compare the efficacy of topical treatments for radiodermatitis
Source: PLoS One. 2019 Jun 10;14(6):e0218018. doi: 10.1371/journal.pone.0218018 (PMC6557505; doi:10.1371/journal.pone.0218018)
Supplement: S1 Attachment — Image calibration and color space conversion from RGB to CIELAB using Matlab, mean L*a*b* calculation. (PDF) [file pone.0218018.s001.pdf]

Data processing for augmented digital image analysis

Step 1. Color calibration using a grey scale reference card

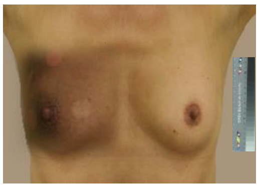

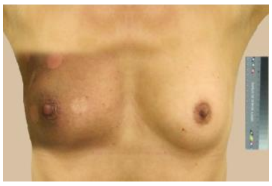

Step 2. Define the areas to be measured and save as separate file

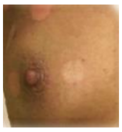

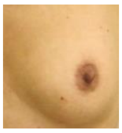

Step 3. Image conversion from RGB color space to the L*a*b* (CIELAB) color space using Matlab (Release 8.1, 2013)

$$\begin{pmatrix} X \\ Y \\ Z \end{pmatrix} = M_{RGB}^{-1} * \begin{pmatrix} R \\ G \\ B \end{pmatrix}$$

with

$$M_{RGB}^{-1} = \begin{pmatrix} 0.412453 & 0.357580 & 0.180423 \\ 0.212671 & 0.715160 & 0.072169 \\ 0.019334 & 0.119193 & 0.950227 \end{pmatrix}$$

ISO13655 includes the current specification of the CIEXYZ to CIELAB conversion as follows:

$$L^* = 116 * Y' - 16$$
$$a^* = 500 * (X' - Y')$$
$$b^* = 200 * (Y' - Z')$$

$$C_{ref} = (X_{ref}, Y_{ref}, Z_{ref})$$

with

$$X' = f_1\left(\frac{X}{X_{ref}}\right), Y' = f_1\left(\frac{Y}{Y_{ref}}\right), Z' = f_1\left(\frac{Z}{Z_{ref}}\right)$$

$$f_1(c) = \begin{cases} c^{\frac{1}{3}} & for\ c > \epsilon \\ \kappa * c + \frac{16}{116} & for\ c \leq \epsilon \end{cases}$$

where

$$\epsilon = \left(\frac{6}{29}\right)^3 = \frac{216}{24389}$$
$$\kappa = \frac{1}{116}\left(\frac{29}{3}\right)^3 = \frac{841}{108}$$

C_{ref} is the reference white point of a specified illuminant. We used the D65 (indirect daylight) with C_{ref} = (0.95047, 1.0, 1.08883) illuminant as a reference.
Available from: Burger W. and Burge M.J.; Digital Image Processing: An algorithmic introduction using java texts in computer science; Springer London, 2016.

Step 4. Calculate mean L*a*b* values of each file using Matlab
